# Modulation of bioactive calcium phosphate micro/nanoparticle size and shape during in situ synthesis of photo-crosslinkable gelatin methacryloyl based nanocomposite hydrogels for 3D bioprinting and tissue engineering

**DOI:** 10.1186/s40824-022-00301-6

**Published:** 2022-10-08

**Authors:** Amitava Bhattacharyya, Gopinathan Janarthanan, Taeyang Kim, Shiva Taheri, Jisun Shin, Jihyeon Kim, Hyun Cheol Bae, Hyuk-Soo Han, Insup Noh

**Affiliations:** 1 Department of Chemical and Biomolecular Engineering, Seoul National University of Science and Technology, Seoul, 01811 Republic of Korea; 2 Convergence Institute of Biomedical Engineering and Biomaterials, Seoul National University of Science and Technology, Seoul, 01811 Republic of Korea; 3 Functional, Innovative and Smart Textiles, PSG Institute of Advanced Studies, Coimbatore, 641004 India; 4 Department of Orthopedic Surgery, Seoul National University College of Medicine, Seoul, 03080 Republic of Korea

**Keywords:** 3D bioprinting, Calcium phosphate, Tissue engineering, Nanoparticles, Nucleation, Site-specific

## Abstract

**Background:**

The gelatin-methacryloyl (GelMA) polymer suffers shape fidelity and structural stability issues during 3D bioprinting for bone tissue engineering while homogeneous mixing of reinforcing nanoparticles is always under debate.

**Method:**

In this study, amorphous calcium phosphates micro/nanoparticles (CNP) incorporated GelMA is synthesized by developing specific sites for gelatin structure-based nucleation and stabilization in a one-pot processing. The process ensures homogenous distribution of CNPs while different concentrations of gelatin control their growth and morphologies. After micro/nanoparticles synthesis in the gelatin matrix, methacrylation is carried out to prepare homogeneously distributed CNP-reinforced gelatin methacryloyl (CNP GelMA) polymer. After synthesis of CNP and CNP GelMA gel, the properties of photo-crosslinked 3D bioprinting scaffolds were compared with those of the conventionally fabricated ones.

**Results:**

The shape (spindle to spherical) and size (1.753 μm to 296 nm) of the micro/nanoparticles in the GelMA matrix are modulated by adjusting the gelatin concentrations during the synthesis. UV cross-linked CNP GelMA (using Irgacure 2955) has significantly improved mechanical (three times compressive strength), 3D printability (160 layers, 2 cm self-standing 3D printed height) and biological properties (cell supportiveness with osteogenic differentiation). The photo-crosslinking becomes faster due to better methacrylation, facilitating continuous 3D bioprinting or printing.

**Conclusion:**

For 3D bioprinting using GelMA like photo cross-linkable polymers, where structural stability and homogeneous control of nanoparticles are major concerns, CNP GelMA is beneficial for even bone tissue regeneration within short period.

**Graphical Abstract:**

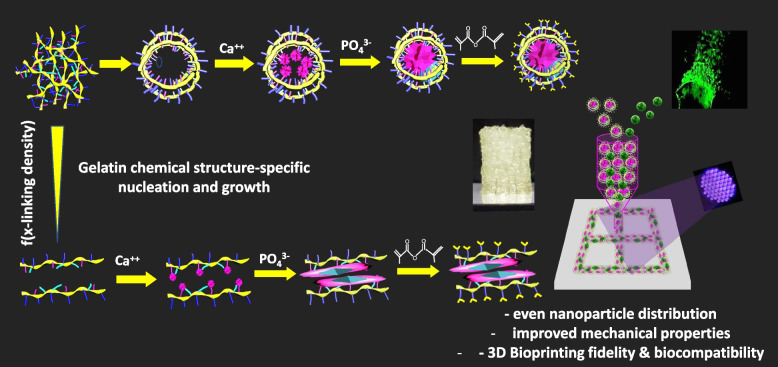

**Supplementary Information:**

The online version contains supplementary material available at 10.1186/s40824-022-00301-6.

## Introduction

The control over tissue regeneration becomes more precise with the use of 3D printing and 3D bioprinting. The cell-laden hydrogel bioink used for 3D bioprinting ensures the control over cell and bioactive molecules distribution throughout the complex 3D printed tissue architecture [1]. The partial methacrylation of gelatin opens a new direction in 3D bioprinting where photo-crosslinking of this unique material leads to biocompatible hydrogel suitable for tissue regeneration. The polymer is known as GelMA where cell encapsulation and subsequent 3D bioprinting with photo crosslinking (adding a photo-crosslinker) is practiced worldwide among the researchers working in this area. However, the printing and mechanical properties of this material limit their use for 3D printing of large and complicated structures [2]. This requires the addition of other polymers or nanofillers to improve its structural stability. Hydroxyapatite micro/nanoparticles formed in photo crosslinked GelMA hydrogel significantly improve its biomechanical properties exhibiting osteogenesis and angiogenesis related differentiation of human umbilical vascular endothelial cells [3]. Thus, most of the bioinks used in advanced research are multicomponent or nanocomposite [4, 5]. For a nanocomposite bioink, the key to achieve strength and structural improvements as well as even tissue regeneration is the homogeneous distribution of nanoparticles in the bioink hydrogel matrix [6]. The controlled distribution of bioactive nanoparticles is expected to contribute in enhanced shape retention ability as well as controlled celll growth and profiferation, leading to even tissue regeneration, throughout the 3D bioprinted architecture as well as controlled distribution of bioactive molecules and particles. Compared to mixed polymer systems, nanoparticles proves more efficient for improving or enhancing the physical and biological properties of the hydrogel matrices during and after 3D printing [7]. Other than printing and structural properties improvements, bioactive nanoparticles activate different cellular activities like attachment, growth, proliferation and extracellular matrix (ECM) secretion leading to tissue formation [8]. They can also participate in differentiation of stem cells. Osteochondral differentiation is intiated by ceramic nanoparticles in stem cells [9, 10]. In spite of all these advantages, the nanoparticle loading in the printable bioink is restricted due to its adverse effects like reduced cross-linking ability of hydrogel matrix, difficulty in controlled dispersion and process ability at higher concentration [11]. Thus, the homogeneous mixing and controlled distribution of nanoparticles in the bioink matrix is a major concern for researchers working in this area [12].

Calcium phosphate nanoparticles are one of the major classes of bioactive nanoparticles widely utilized for 3D bioprinting in skeletal tissue engineering [13, 14]. They are the major constituents of bone ECM for strengthening bones [15]. The biphasic calcium phosphate nanoparticles was found to enhance osteogenesis and hard tissue regeneration such as bone [16]. Other than up-regulating osteogenic markers, the addition of calcium phosphate nanoparticles has been widely practised in bone tissue engineering for their ability to improve mechanical properties of polymer scaffolds near to native bone tissue [17]. However, nonuniform mixing can lead to failure of structures which restricts its use at higher doses. Most widely used incorporation of calcium phosphate particles in hydrogel adopted by researchers is conventional direct mixing where nanoparticles are dispersed in the polymer matrix before gel formation or crosslinking reaction. This helps to distribute the nanoparticles better in low viscous system and subsequent crosslinking traps the nanoparticles in the hydrogel matrix. The movement restriction helps to avoid agglomeration. This mixing process often leads to inhomogeneity in the bioink matrix and the the structure suffers with non-uniform mechanical and structural properties [18]. Other than this, in situ nanoparticle formation [19] and biomimetic (in vivo*/*in vitro) mineralization [20] are adopted by researchers. The byproducts formed during the nanoparticle formation process cannot be removed in these processes. Furtrher, biomimetic mineralization is a slow process and thus the initial shape retention ability of the 3D printed structure should be adequate to support it till the process matures. The dual cross-linking of mixed polymer systems facilitates steady increase in viscosity and cross-linking density, and reduces the chance for cell sedimentation confining them in the polymer network [21]. In methods like printing-then-mixing, dispersion can be better controlled and high cell viability is achieved [22]. However, it is difficult to control this process. Further, the porosity of the gel scaffold is significantly reduced by the in situ nanoparticle formation, though it has better dispersion uniformity than direct mixing [23]. Homogeneity in crosslinking is also affected and the cell penetration and growth throughout the scaffold is restricted due to reduced porosity.

**Fig. 1:**
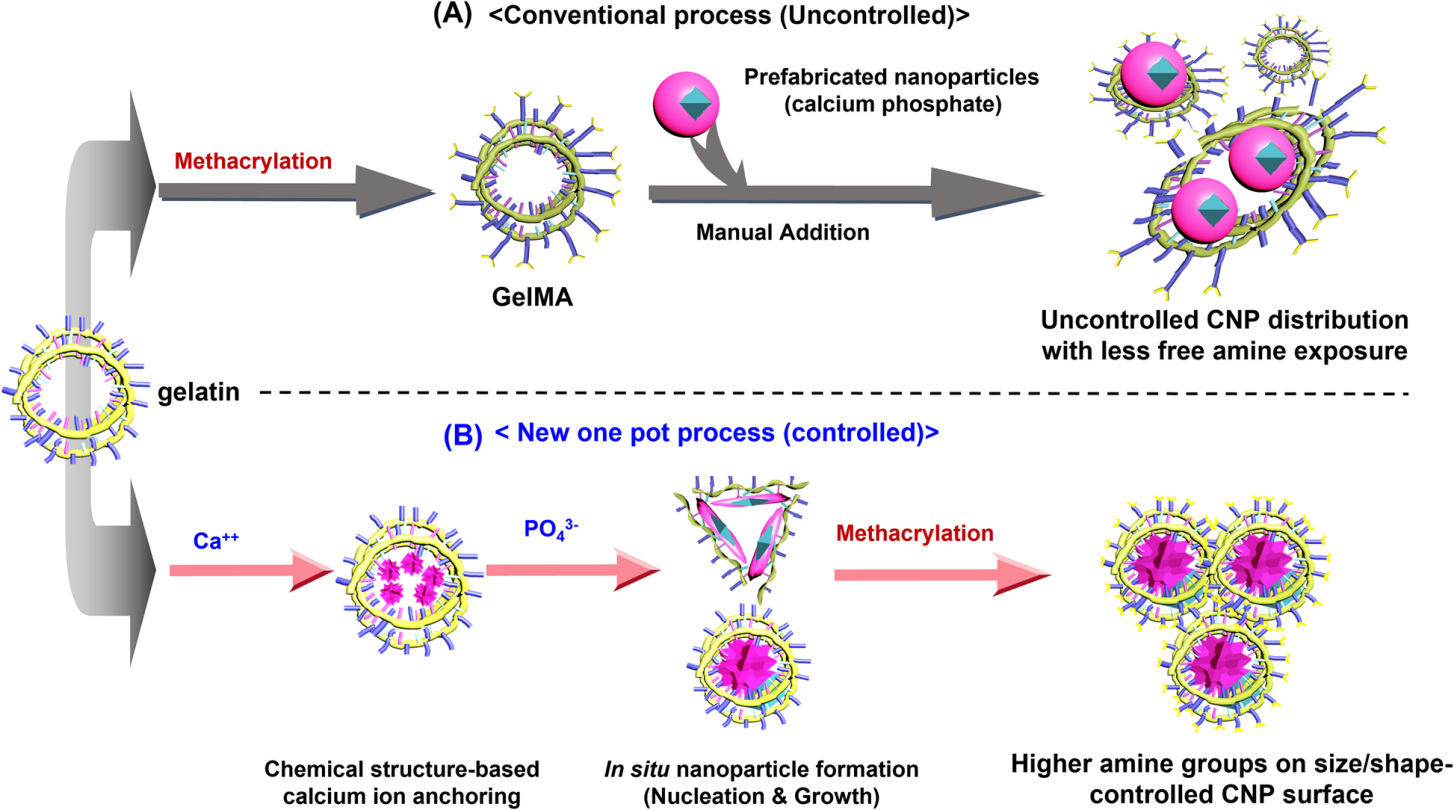
Synthesis of calcium phosphate nanoparticle (CNP) reinforced gelatin methacryloyl. **A** A conventional nanoparticle dispersed gelatin methacryloyl polymer synthesis process and (B) newly developed gelatin structure-based in situ one pot method with higher free amine groups exposed outside

In this work, a novel technique is introduced as below to ensure controlled bioactive micro/nanoparticle distribution via gelatin structure based nucleation and particle growth to reinforce the GelMA, potentially leading to better 3D bioprintability and bone tissue regeneration [24]. Figure 1 depicts the scheme of synthesis of such material through one pot processing by comparing with that of conventioal mixing methods. Amorphous calcium phosphate micro/nanoparticles have been prepared with gelatin as stabilizing agent before the methacrylation reaction. The advantage of this gelatin structure-based nanoparticle formation over conventional mixing of nanoparticles is the control of the expected homogeneity, chemical structure-based distribution and probable size by utilizing scientific understanding of gelatin structure and polymer conditions. The higher interaction between negatively charged groups of gelatin such carboxyl and hydroxyl than amine with Ca^++^ is expected to build a conformational change in the polymer chain leading to higher availability of amines on the surface of the coated gelatin on the synthesized nanoparticles. The size and shape of the synthesized nanoparticles are controlled by changing the stabilizer (gelatin) content. This techique opens an immense possibility for in situ synthesis of various nanostructured nanocomposite hydrogels. Thus produced micro/nanoparticle reinforced GelMA polymers and crosslinked hydrogels are studied for its mechanical and biological properties for its suitability in 3D extrusion-based bioprinting and tissue engineering such as bone.

## Experimental

### Methods

Pure gelatin methacryloyl (GelMA) polymer was synthesized by adding 1 g gelatin (from bovine skin, ~ 75 bloom, G6650, Sigma Aldrich, USA) and 50 ml phosphate buffered saline (PBS) to the 100 ml 2-sided round bottom flask in an oil bath. It was stirred at 50 ^o^ C at 400 rpm for 12 h to dissolve sufficiently. The temperature was increased to 60 ^o^ C and stir for 30 min. The pH is adjusted to 7.6 with a small amount of 5 M NaOH solution. 4 ml methacrylic anhydride (MA, Molecular Weight: 154.16, 276,685, Sigma Aldrich, USA) was added slowly drop by drop and stirred for 3 h. 50 ml PBS was added in the reaction flask and stirred for 5 min. Then, it was allowed to cool down at room temperature. The size and shape of nanoparticles depends on the amount of gelatin used during the synthesis reaction.

Considering the size distributions, 1 g gelatin was selected for nanoparticle reinforced GelMA (CNP GelMA and CNP (50%) GelMA) polymer synthesis. 10 mM CaCl_2_ (0.11 g) is dissolved in 50 ml distilled water. 1 g gelatin was added to it and stirred in oil bath at 50 ^o^ C at 400 rpm for 6 h to dissolve completely. pH is adjusted to 8 using NaOH. 0.0852 g Na_2_HPO_4_ is dissolved in 10 ml distilled water separately and added with stirring for 2 h. The temperature is increased to 60 °C and continue stirring for 30 min 4 ml methacrylic anhydride (MA, Molecular Weight: 154.16, 276,685, Sigma Aldrich, USA) was added slowly drop by drop and stirring continued for 3 h. 40 ml PBS was added in the reaction flask with stirring for 5 min and allowed to cool down. After reaction, the obtained compounds were dialyzed in 5 L distilled water for 4 days (molecular porous membrane tubing molecular weight cut off 6 ~ 8KD; Standard RC Tubing, Part Number 132665, Spectrum Laboratories, Inc, USA). Then the samples were lyophilized for 4 days.

Micro/nanoparticle (CNP) was synthesized using the same protocol described above except the methacrylation step. After synthesis of gelation stabilized micro/nanoparticle, repeated wash with hot deionized (DI) water and vacuum filtering were carried out with 0.1 µm acetate filter. Finally, it was dried at 60 ^o^ C and grinded to fine powder using mortar and pestle to use as CNP. For CNP + GelMA sample, CNP was taken as 20% (w/w) to mix with GelMA in DI water with 2 h vortex shaking and 30 min ultrasonic treatment. The amount is equivalent to CNP GelMA.

Hydrogels were obtained by crosslinking all synthesized photo cross-linkable gelatin-based polymers (GelMA, CNP (50%) GelMA, CNP GelMA and CNP + GelMA) by using 5% (w/w) Irgacure 2955 as crosslinker with UV irradiation.

### Characterizations

#### Morphological and functional groups

The surface morphology and energy dispersive x ray (EDS) were examined by SEM (Model: TESCAN VEGA3, Czech Republic). All samples for SEM were prepared and dried using lyophilization or freeze drying, and sputter coated with platinum. The structural features and selected area electron diffraction (SAED) pattern were examined using a Transmission electron microscope (TEM, JEOL, Japan) at an accelerated voltage of 100 kV. For particle size analysis, dynamic light scattering from Malvern Inc. UK, was used.

For quantification of the remaining free amino groups in the synthesized photo cross-linkable nanoparticle dispersed and pure gelatin methacryloyl polymer samples, 2,4,6-Trinitrobenzenesulfonic acid (TNBS) assay was carried out [25]. For functional group analysis, samples were dried properly and recorded using an attenuated total reflectance ATR-FTIR spectrometer (model: Travel IR, Smiths Detection, USA) in 500 ~ 4000 cm^−1^ range. ATR is used to measure FTIR spectra for direct analysis of dried samples without making pellet. Dried powdered samples were used for XRD spectra recording (Bruker D8 Advance) with Cu Kα radiation (λ = 0.154 nm), 2θ ranging from 2° to 70° (interval of 0.02°) and an operating voltage of 40 kV and current of 30 mA. Thermogravimetric analysis (TGA) was also performed with a thermogravimetric analyzer (TGA-50 series, Shimadzu; Japan) at 10 °C/min under argon atmosphere till 750 °C. Nuclear magnetic resonance (NMR) imaging was performed for gelatin and GelMA polymer (700 MHz nuclear magnetic resonance (NMR) spectrometer, DD2700, Agilent Technologies, USA) in DMSO-*d*6 and the spectrum was analyzed using MestReNova software (version 12.0.2–20,910, 2018).

#### Rheology, mechanical and 3D printing

The rheological properties of the different hydrogels were examined using an R/S plus rheometer (Brookfield, USA, 25 mm diameter two rotating circular disc plates parallel to each other with a 1 mm gap) at 25 °C and the shear rate was applied from 1 to 100 s^−1^ for 100 s. A texture analyzer analysis instrument (Stable Micro Systems model, TA.XT plus, Surrey, UK) was used to determine the printing-related mechanical properties of the cylindrical gel samples (10 mm height and 9 mm diameter) in compressive strain mode at a speed of 1 mm/min at 25 °C. Swelling study was carried out in phosphate buffer saline (PBS) with lyophilized gel samples. The weight gain percentage with time is recorded.

A customized 3D bioprinter (SeoulTech, Korea) was used for 3D printing and 3D bioprinting [26]. The 3D lattice structure was designed using Solid Works software (Dassault Systems SolidWorks Corp, USA), and the G-codes for the STL files were generated using a slicing software (Simplify 3D version 4.0, USA), as described previously [27]. The GelMA (3 ml) mixed with 5% (w/w) Irgacure 2955 as photo crosslinker was loaded inside the plastic syringe (5 ml, Musashi Engineering Inc., Korea) attached with a needle (23 gauge). The syringe needle was placed proximal to the stage with the substrate by adjusting the Z-axis (syringe holder), X and Y axis (stage) using the software. A printing speed of 150 mm/min with pressure of 50 kPa at a temperature 27 °C was used for 3D (bio) printing. GelMA hydrogel was obtained by crosslinking of GelMA (and 5% (w/w) Irgacure 2955 mixture) with continuous UV irradiation on the printed structure during the printing process.

#### Cell culture studies

For cell culture experiments, two different cells were used, MC3T3 and adipose tissue derived mesenchymal stem cell(AdMSC). An osteoblast cell line derived from mouse calvaria (Passage 13, MC3T3-E1 cell line, Young Science Inc., Korea) was sub-cultured in a 24-well plate with α-Minimum essential media (MEM) (Sigma Aldrich, USA) containing 10% fetal bovine serum (Gibco Korea, Korea) and penicillin–streptomycin (100 unit/mL) in CO_2_ incubator (with 5% CO_2_ at 37 °C). The different crosslinked gel samples were prepared carefully under sterile conditions, and the gels were washed thoroughly with phosphate buffer saline (PBS) and medium to avoid any contamination. MTT (3-(4,5-dimethylthiazol-2-yl)-2,5-diphenyl tetrazolium bromide) assay was carried out using extraction protocol as described previously [28]. 10 mg of crosslinked hydrogel samples were soaked for 48 h in 1 mL of DMEM cell culture medium and incubated at standard conditions to obtain the hydrogel extracts for the MTT assay. MC3T3 (passage 13) cells were seeded on to the 96 well plate with 1000 cells/well (100 µL DMEM/well). The medium was replaced with the extract liquid and incubated at standard cell culture conditions and the samples were assessed for cell viability at day 1, 2 and 3 using MTT assay. The in vitro cell culture study was performed for up to 7 days on 3D bioprinted (cell embedded) photo crosslinked gel samples, and the samples were observed on days 0, 3, and 7. MC3T3 cells (0.5 × 10^6^/mL) was mixed with 30% (w/v) sterilized sample solutions (and 5% (w/w) Irgacure 2955 mixture) and UV crosslinked during printing. Live/dead staining (LnD) was carried out using the LIVE/DEAD ® Viability/Cytotoxicity Kit (Invitrogen, USA), which combines green, fluorescent calcein-AM staining to show intracellular esterase activity with red-fluorescent ethidium homodimer-1 staining to confirm the loss of plasma membrane integrity. The images of the live and dead cells were taken after the addition of 1.2 μL of 2 mM ethidium homodimer-1 (EthD-1) and 0.3 μL of 4 mM calcein AM into 600 μL of phosphate-buffered saline (PBS). After the addition of the two agents, the plate was incubated in the dark for 30 min. The live and dead cells were captured using different filters present in the fluorescence microscope (Leica DMLB, Germany) and merged using the LAS-X Leica microsystems software.

Adipose derived mesenchymal stem cells (AdMSC, *p* = 7) was used to create bioprinted structures using the synthesized gels. 0.5 million cells/ml were used to encapsulate with 30% (w/v) sterilized GelMA, CNP + GelMA and CNP GelMA with 5% (w/w) Irgacure 2955 for photo crosslinking (5 min UV exposure). LnD for day 0, 3 and 7 were carried out. Standard protocols given by the suppliers was followed for the hematoxylin and eosin (H & E) staining and alkaline phosphatase (ALP) assay (absorbance at 405 nm) at the end of Day 7.

The gene expressions of osteogenesis-related markers (collagen type I (Col-1), osteocalcin (OCN) and ALP) and mechanosensing-related marker (focal adhesion kinase (FAK) is measured using quantitative reverse transcription-polymerase chain reaction (qRT-PCR) with different sample treated cells at the end of day 8. Sample solutions (2 ml) and 5% (w/w) Irgacure 2955 mixture were added for number of cell culture plates with diameter 100 mm. It was crosslinked and UV sterilized for 5 h under biosafety cabinet UV light to form different film like crosslinked gel samples (0.25 mm thickness) on the cell culture plates. AdMSCs are seeded onto these plates at a density of 5 × 10^3^ cells/plate and cultured. At the end of the 8 days, cells in CNP GelMA hydrogel-based plates attain 100% confluency. Hence, to keep the conditions similar all the cells are collected from each group and freeze to transfer for RNA extraction.

The total RNA is extracted using Total RNA Kit (Omega, Hong Kong). 1 μg of total RNA is reverse transcribed to complementary DNA (cDNA) using the PrimeScript RT Master Mix Kit (Takarabio, Hong Kong). qRT-PCR shall be performed by the Real-Time PCR Detection System with TB Green Premix Ex Taq Kit (Takarabio, Hong Kong) under the conditions of 5 s at 95° C, 30 s at 60° C, and the fluorescence intensity was recorded for 40 cycles. The Livac method (2^−ΔΔC^_T_) was adopted for quantification of the relative expression of each interested gene, and GAPDH as the housekeeping gene was used as the internal control [29]. The primer sequences of each gene utilized for PCR amplification were listed in Supplementary Table 1.

#### Statistical analysis

All images were analyzed using ImageJ software (NIH, USA) to estimate pore sizes and live/dead or cellular spreading on the gel surface. Statistical analysis was performed by one-way analysis of variance with 95% confidence (*p* ≤ 0.05) using the Tukey’s Means Comparison Test with Origin 9.0 software. The process significance tests were carried out with the null hypothesis “there is no significant difference between the pore X created in different lyophilized cross-linked samples”, where X is the diameter and number per unit area.

## Results

### Morphology, shape, and size distributions of the synthesized micro/nanoparticles

**Fig. 2:**
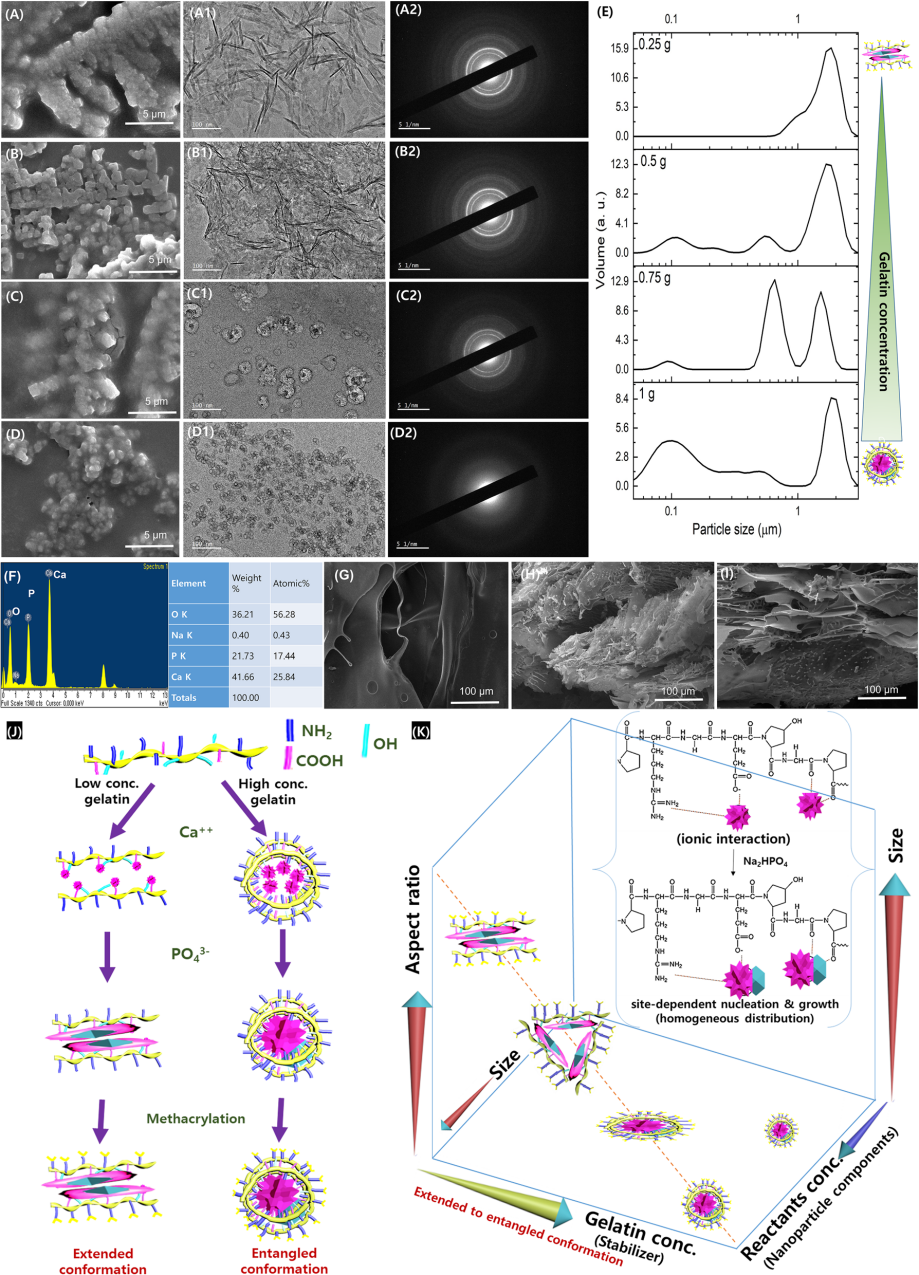
Size and shape modulation of synthesized calcium phosphate nanoparticles (CNP) with the increase in stabilizing agent (Gelatin) during the synthesis process: (**A**) with 0.25 g gelatin, (**B**) with 0.5 g gelatin, (**C**) with 0.75 g gelatin, and (**D**) with 1 g gelatin; (**E**) corresponding particle size distribution (1.753 μm < 50% for 0.25 g; 1.576 μm < 50% for 0.5 g; 0.787 μm < 50% for 0.75 g; 0.296 μm < 50% for 1.0 g), (**F**) EDS showing Ca, P and O as major components, confirming formation of calcium phosphate nanoparticles; SEM images of cross-linked hydrogel (**G**) GelMA, (h) CNP (50%) GelMA, (**I**) CNP GelMA (solutions of 30% w/v were cross-linked with 5% w/w Irgacure 2955 for 5 min UV exposure), (**J**) scheme for size and shape modulation, depending on gelatin concentration and their aspect ratios with possible nucleation development

The morphology, shape, and size distributions of the synthesized micro/nanoparticles and hydrogels are shown in Fig. 2. The EDS and SAED pattern confirmed the synthesis of calcium phosphate micro/nanoparticles in different amount of gelatin matrix (Fig. 2A and A1, B and B1, C and C1, D and D1 for 0.25 g, 0.5 g, 0.75 g and 1.0 g gelatin, respectively, in SEM and TEM, respectively). The rings observed in selected area electron diffraction (SAED) pattern in Fig. 2 (A2, B2, C2 and D2) indicate formation of the calcium phosphate micro/nanoparticles. Rings at 0.3547 nm and 0.2857 nm are distinct, expected XRD peak around 26 deg. (002) and 32 deg. (211), respectively. Ring around 0.1743 nm indicate expected small peak around 50 deg. 2θ (213) in XRD. The amount of gelatin used for stabilizing the structure of nanoparticles has substantial influence on their particle size distribution (Fig. 2E). With the increase in gelatin concentrations the synthesized particle sizes become smaller and more particles are on lower side in dimension. Both 0.25 and 0.5 g gelatin used for stabilizing the synthesized micro/nanoparticles showed that most of the particles are more than 1.5 µm. With 1 g gelatin, more than 50% micro/nanoparticles’ sizes are lower than 293 nm, while 787 nm is the median value for 0.75 g gelatin stabilized sample. TEM images revealed that the synthesized particles are spindle-shaped (Fig. 2A) when less amount of gelatin was used as stabilizing agent. If the gelatin content is increased in the reaction media, the shape of the synthesized nanoparticle is gradually converted to spherical shape with significant decrease in size (Fig. 2D).

The EDS result confirms the presence of pure calcium phosphates (Fig. 2F) with very low impurity of sodium (Na: 0.4%). More washing should remove the small amount of Na ion present as a byproduct of the synthesis reaction. For subsequent experiments, 1 g gelatin-based samples were used. SEM images of GelMA, CNP (50%) GelMA and CNP GelMA (Fig. 2G–I) showed the nanoparticles formed in the GelMa.

### Chemical structural changes in gelatin after the nanoparticle synthesis and methacrylation

**Fig. 3:**
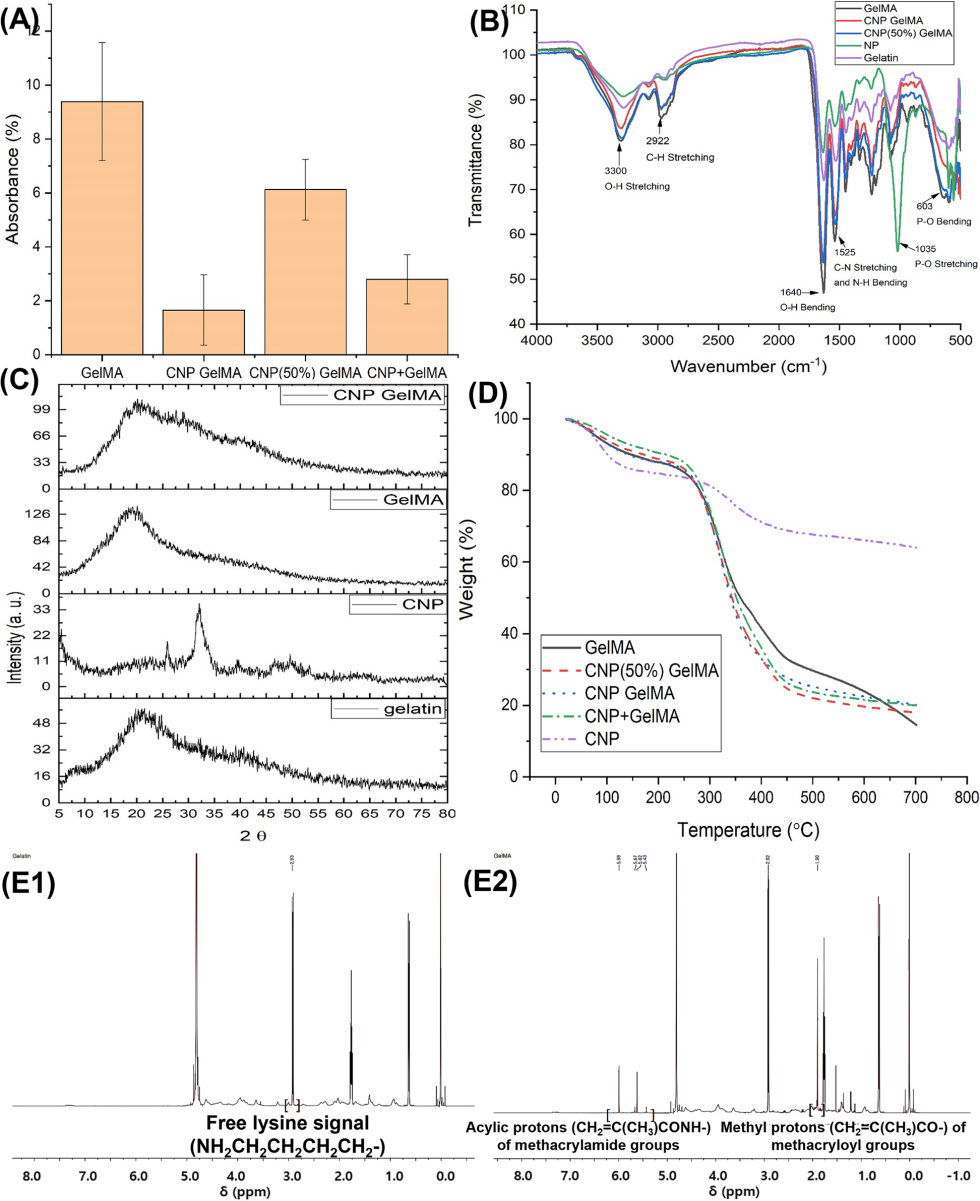
Analysis of CNP-gelatin-methacryloyl polymer samples, where (**A**) TNBS assay to estimate amount of free amino groups in the synthesized polymers (free amine groups present in pure gelatin was considered as 100%), (**B**) FTIR showing the changes in functional groups; (**C**) XRD of synthesized nanoparticle (CNP) and CNP GelMA, (**D**) TGA for the synthesized gels, (E-1,2) NMR spectra showing methacrylation of gelatin

Figure 3 shows the changes in chemical structures of the gelatin after the synthesis procedures. The TNBS assay measuring the amount of free amine groups confirms that the methacrylation is better for our newly designed synthesis protocol to that of conventional GelMA synthesis (Fig. 3A, CNP GelMA *vs*. CNP + GelMa or GelMA). FTIR of synthesized nanoparticles shows phosphate (PO_4_)^3−^ related bands at 564, 603, 963, 1035, and 1094 cm^−1^, carbonate (CO_3_)^2−^ bands at 1567 and 2300–2500 cm^−1^, and broad peak in between 3000 and 3800 cm^−1^ for O–H group of adsorbed water (Fig. 3B). In the FTIR of CNP GelMA, amide peaks of GelMA are visible. The phosphate peaks’ intensities around 1000 – 500 cm^−1^are increased with increase in calcium phosphates in GelMA.

In the XRD of synthesized nanoparticles, GelMA and CNP GelMA (Fig. 3C), the nanoparticles were identified as amorphous calcium phosphate: JCPDS file # 01–080-3958, (002) around 26 deg. and (211) around 32 deg. As the amount of CNP is low compared to gelatin, after CNP inclusion a small hump is observed in the XRD spectra of the CNP GelMA in these diffraction angles. Figure 3D shows the TGA curves of the synthesized samples. The amount of micro/nanoparticles in CNP GelMA is comparable to that of the separately mixed ones (CNP + GelMA) as the residual mass is same in both cases. The residual mass of CNP (50%) GelMA shows the evidence of nanoparticle formation in reduced quantity. For pure nanoparticle (CNP), some mass loss is observed at higher temperature due to removal of stabilizing agent (Gelatin) and water. From the NMR study (Fig. 3E1 & E2), the successful methacrylation of gelatin is evident by observing free amine and methacryloyl [30].

### Rheological and print-related mechanical properties

**Fig. 4:**
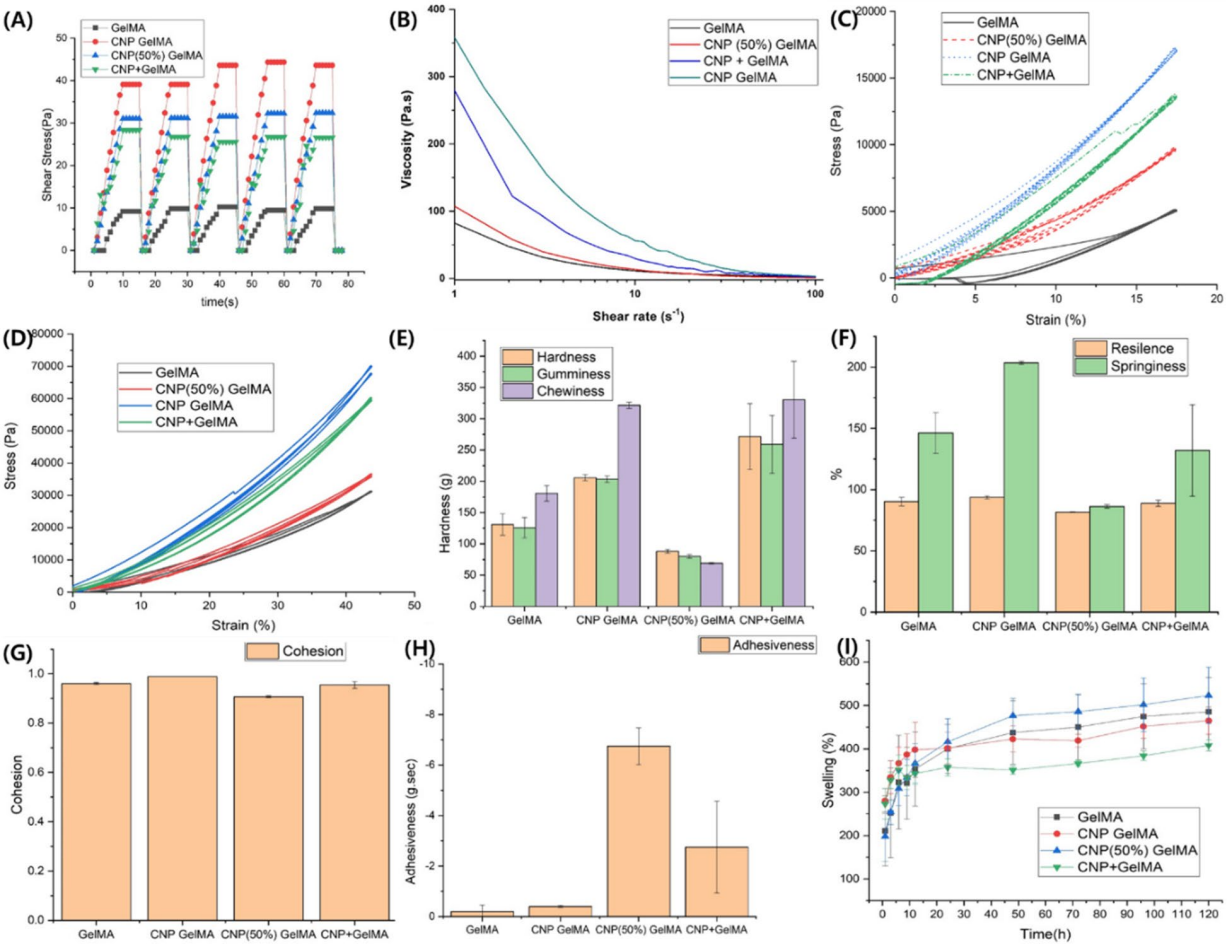
Rheological behaviors of the cross-linked gels (**A**) cyclic rheology test with 60 s UV exposed, (**B**) rheology test with 330 s UV exposed; cyclic compressive responses of the cylindrical crosslinked gel samples (H = 10 mm, D = 9 mm) (**C**) 5 cycle with 17.5% compression and (**D**) 2 cycle with 43.75% compression; (**E**–**H**) mechanical texture properties related to 3D printing, and (I) swelling of different gels in PBS. In CNP + GelMA, CNP was taken as 20% (w/w) to mix with GelMA which is equivalent to synthesized CNP-GelMA. 30% w/v of all solutions are crosslinked with Igacure 2955 (5% w/w). For Figure D-I, 5 min UV exposure was given for crosslinking

Figure 4 explained the rheological and print-related mechanical properties of the different gels synthesized in this work. The CNP GelMA showed better cyclic rheology value within 60 s of UV exposure (Fig. 4A). It has better viscosity when cross-linked for 330 s (Fig. 4B). CNP GelMA shows more than 3.5 times increase in viscosity at 1 Hz shear rate than pure GelMA. The shear-thinning property of GelMA retains for all samples even after inclusion of nanoparticles. The viscosity build-up for different gels at different time of UV crosslinking is shown in Supplementary Fig. 1 over a shear rate of 1 ~ 100 s^−1^. The cross-linking ability of CNP GelMA is remarkably better than pure GelMA. Within 210 s of UV exposure, it attains good viscous property while pure GelMA attains its maximum in 270 s.

The fully cross-linked CNP GelMA hydrogel samples showed better cyclic mechanical as well as printing properties (Fig. 4C-H). From the five-cycle compressive test (Fig. 4C), it is evident that the nanoparticles improve the cyclic compressive strength more than three times than the cross-linked pure GelMA hydrogel. After five cycles of compressive loading, only CNP GelMA hydrogel has shown 100% strength recovery (other are around 98.5%). In two-cycle compressive test with more than 40% compression, CNP GelMA hydrogel has shown more structural retention possibility than the conventionally mixed CNP with GelMA (CNP + GelMA hydrogel). Separately mixed CNP also improves its cyclic compressive strength, however, the springiness of CNP GelMA hydrogel is much better than CNP + GelMA hydrogel, indicating its better recovery from cyclic strain (Fig. 4D). Springiness is the ability of the material to return to its original shape. The large variation in hardness, gumminess, chewiness, and springiness values for CNP + GelMA hydrogel indicates non uniformity due to mixing. The parameters like gumminess and chewiness are for crunching [31]. No significant variation is observed in terms of cohesiveness among the gel samples, indicating that the strengths of the gels in holding their own structures are almost equally good for all synthesized gels (Fig. 4G). Adhesiveness is the ability to adhere to other surfaces. Improved adhesiveness of both CNP (50%) GelMA and CNP GelMA helps to stick the printed structure on the surface of its substrate and reduces the chance of displacement during multi-layer printing process (Fig. 4H). Resilience is a measure of energy loss during cyclic deformation which is also comparable for all the gels. The swelling percentage is significantly reduced using CNP GelMA hydrogel, though CNP + GelMA hydrogel showed higher swelling (Fig. 4I).

### 3D printing performance of different synthesized hydrogels

**Fig. 5:**
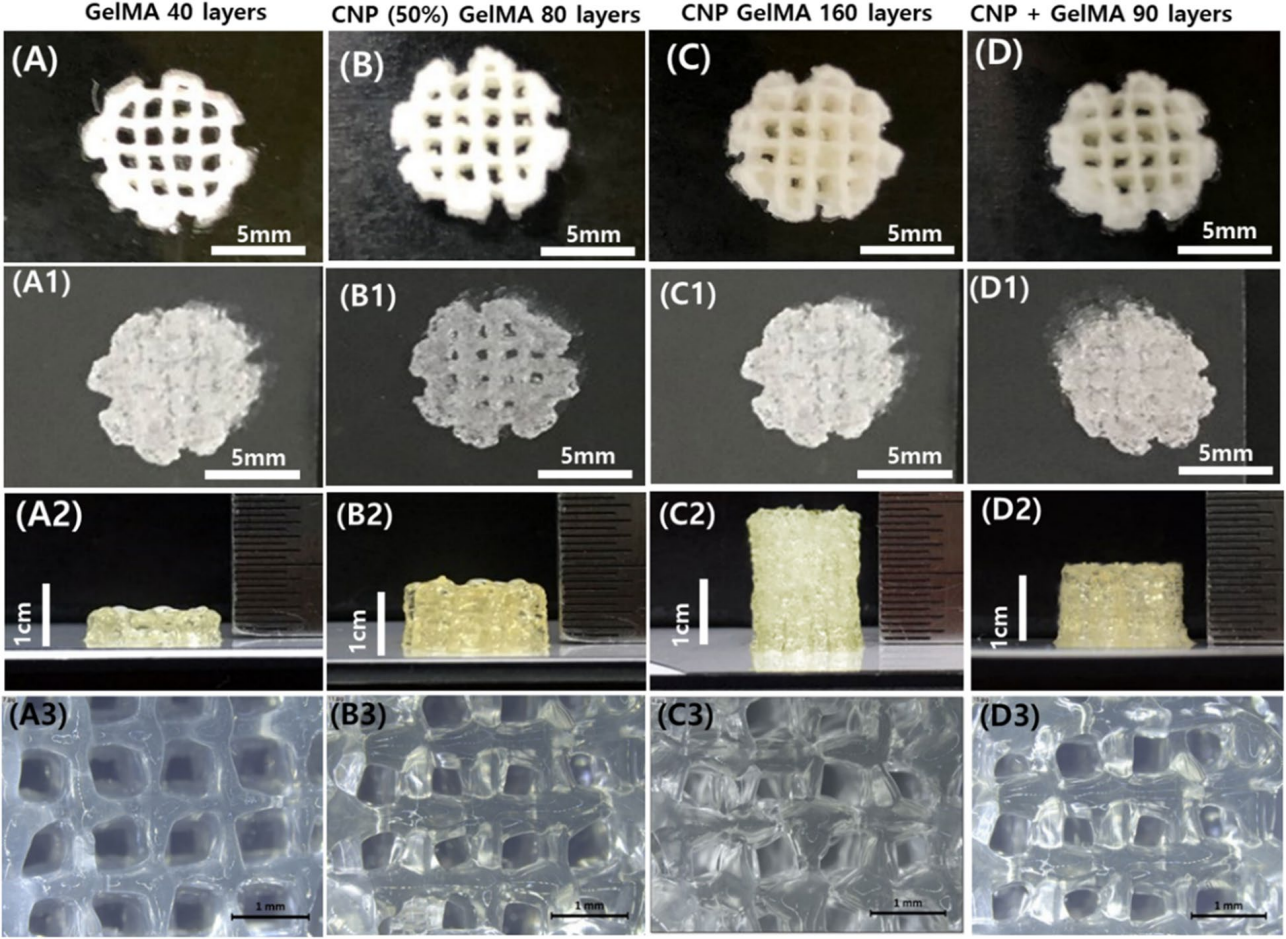
3D printing of (**A**) GelMA (40 layers printed, beyond that structure started distorting), (**B**) CNP (50%) GelMA (80 layers printed, beyond that structure started distorting), (**C**) CNP GelMA (160 layers printed, beyond that structure started distorting), (D) CNP + GelMA (90 layers printed, beyond that structure started distorting). 40% (w/v) sample solutions in DI water were used with 5% (w/w) Irgacure 2955 as a cross-linker, and UV light was continuously supplied on the printed structure during the printing process as shown in supplementary video

Figure 5 shows the 3D printing performance of different synthesized hydrogels. A porous cylindrical structure was selected for this experiment. Supplementary video file is supplied to understand the printing process. The heights of the printed samples are shown in the Fig. 5. The maximum number of layers for GelMA is 40 (Fig. 5A). After that the structure stared distorting. The maximum number of layers is a significant parameter for printing performance quantification. With CNP (50%) GelMA and CNP GelMA, 80 and 160 layers of printing (Fig. 5-B, C) are achieved, respectively, while CNP + GelMA can successfully printed up to 90 layers (Fig. 5D).

### Structural morphology of 3D printed lyophilized samples

**Fig. 6:**
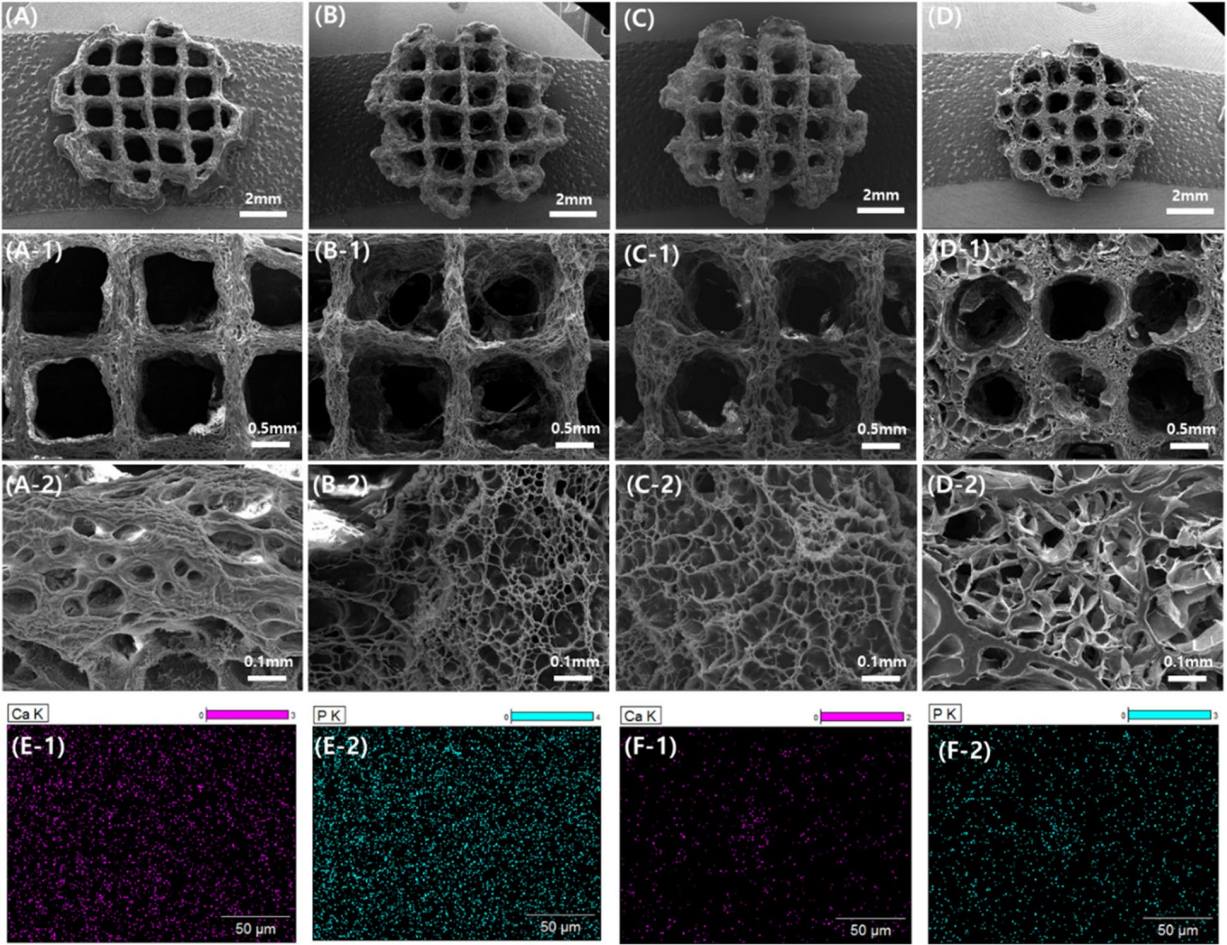
SEM images of the lyophilized 3D printed samples at different magnifications, showing the interconnected porous cross-linked gel structure, (**A**) GelMA, (**B**) CNP (50%) GelMA, (**C**) CNP GelMA, (**D**) CNP + GelMA, EDS mapping for CNP GelMA (E-1) Ca, (E2) P; CNP + GelMA (F-1) Ca, (F-2) P. For crosslinking of solutions, continuous UV irradiation was given to the 40%(w/v) polymer solutions in DI water with Igacure 2955 (5%, w/w)

Figure 6 shows the lyophilized 3D printed samples. At higher magnifications, the porous structures and interconnectivity of pores are visible for all cross-linked samples. However, cross-linked GelMA and CNP + GelMA hydrogels suffer with a smaller number of larger pores (Fig. 6A and D). Using ImageJ software, most of the pore sizes are observed in the range of 40 ~ 100 μm for GelMA and CNP + GelMA hydrogels. The number of pores per unit area is also significantly less than both CNP GelMA and CNP (50%) GelMA cross-linked hydrogels. The synthesis process of CNP GelMA described in this work resulted in uniform pore distribution and smaller size pores with less variation in diameter. Lyophilized crosslinked CNP (50%) GelMA hydrogel has smallest pore sizes (5 μm) but larger variations (5 ~ 40 μm) than lyophilized CNP GelMA crosslinked hydrogels (most of the pores are 10 ~ 20 μm). The statistical significance test concludes with 95% confidence level that the gelatin structure-based CNP-nucleated crosslinked hydrogels produced through our process is significantly better than the conventional GelMA and CNP + GelMA crosslinked hydrogels for creating a greater number of small pores with lesser variation in pore sizes. Stepwise elimination of sample data indicates that both CNP GelMA and CNP (50%) GelMA hydrogels can create better interconnected porous structure than the CNP + GelMA hydrogels. Further, Ca and P mappings in EDS for crosslinked CNP GelMA and CNP + GelMA hydrogels indicate homogeneous distribution of the synthesized CNPs in the case of CNP GelMA hydrogel (Fig. 6E) which is missing for CNP + GelMA hydrogel (Fig. 6F).

### Cell culture experiments with osteoblast cells

**Fig. 7:**
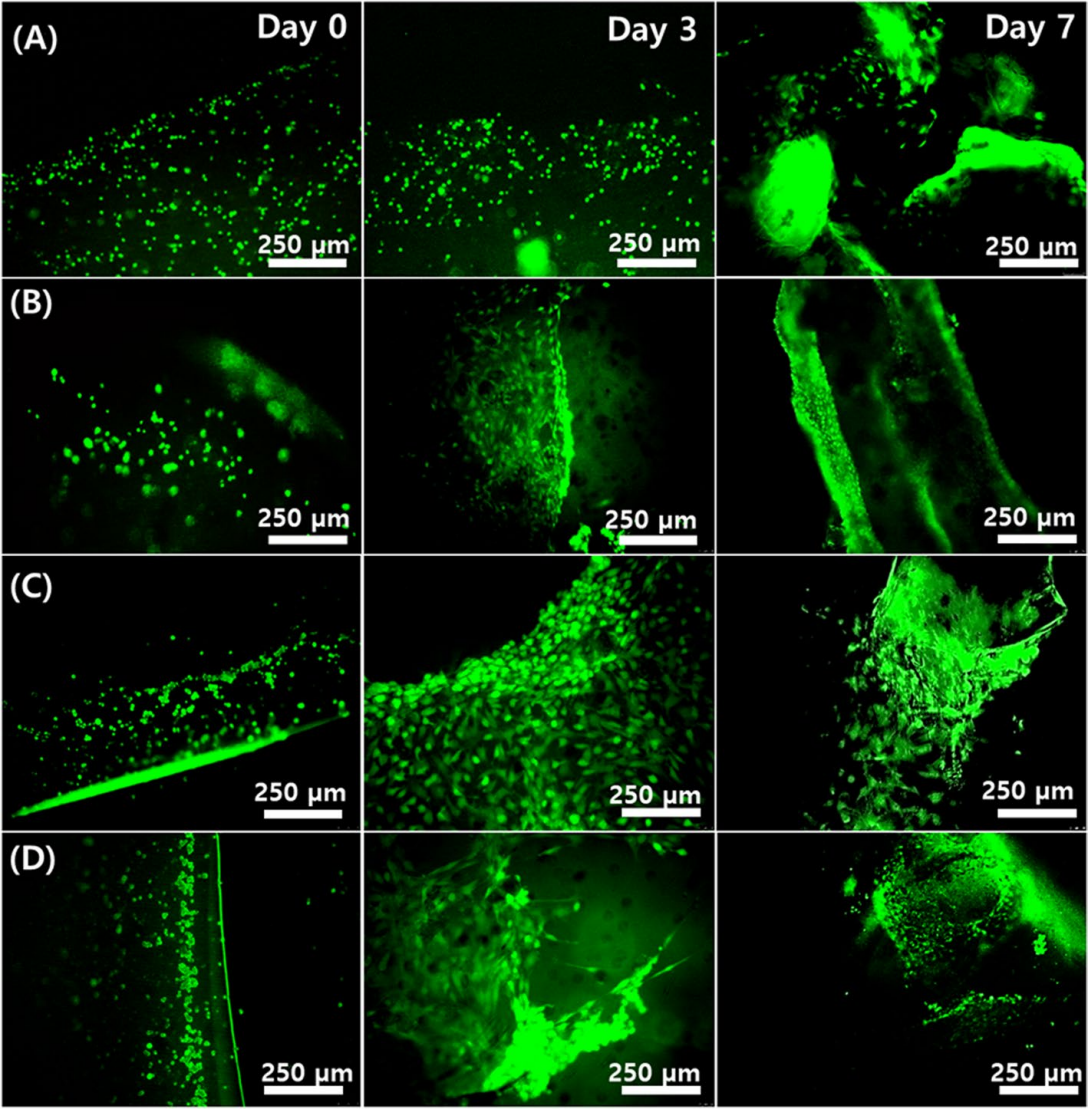
Live and dead assay of the cell encapsulated crosslinked hydrogel samples at Day 0, 3 and 7: (**A**) GelMA, (**B**) CNP (50%) GelMA, (**C**) CNP GelMA (**D**) CNP + GelMA, all samples (30% w/v) are mixed with Irgacure 2955 (5% w/w) and cross-linked with UV exposure during bioprinting. 0.5 million/ml MC3T3 cells were mixed with GelMA/Irgacure solution and bioprinted before crosslinking (UV treatment)

Figure 7 shows the cell culture results (live/dead assay) for the 3D bioprinted cylindrical structures with MC3T3 mouse osteoblast cells. All CNP-reinforced crosslinked hydrogels showed more cell proliferation at the end of 7 days. However, the inhomogeneous distribution/dispersion of micro/nano-particles (CNP) in the GelMA matrix (CNP + GelMA) resulted in the uneven growth and proliferation of cells in the structure (Fig. 7D). Both the CNP (50%) GelMA and CNP GelMA hydrogels showed even distribution of cells throughout the structures which cover completely within 7 days (Fig. 7B and C). In the scanned area, the number of live cells is more than 4 times than the pure GelMA (Fig. 7A).

### Cell culture experiments with AdMSC cells

**Fig. 8:**
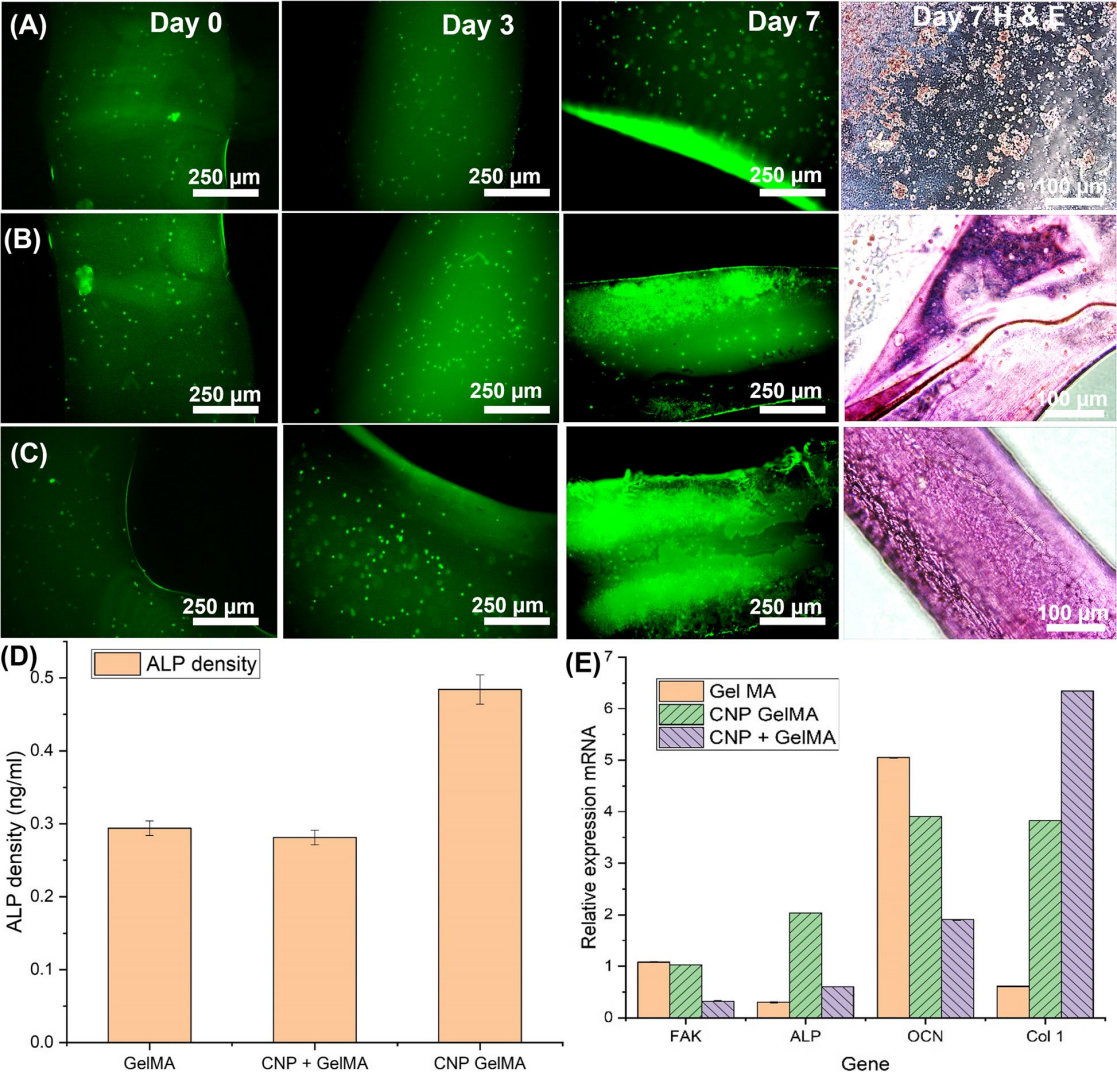
Live and dead assay of the cell-encapsulated cross-linked hydrogel samples at Day 0, 3 and 7 and H & E Day 7: (**A**) GelMA, (**B**) CNP + GelMA, (**C**) CNP GelMA, all samples (30% w/v) are mixed with Irgacure 2955 (5% w/w) and cross-linked with UV exposure during bioprinting. 0.5 million/ml AdMSC cells were mixed with GelMA/Irgacure solution and bioprinted before crosslinking (UV treatment). **D** ALP activity, (**E**) Gene expression study (RT-µPCR Day 8)

Figure 8A–C shows the LnD and H & E for bioprinted structures with AdMSC cells. With AdMSC cells also, even cell proliferation and growth were observed in photo crosslinked CNP GelMA hydrogel samples. The cells encapsulated in cross-linked GelMA hydrogel did not show substantial growth, only discreet cells were observed in L&D and H & E staining even at the end of 7 days (Fig. 8A). Cross-linked CNP + GelMA hydrogel sample shows evidence of tissue formation, but the cells are grown as a cluster after day 7(Fig. 8B). The cells grown on the CNP GelMA covered the entire surface uniformly with dense packing as observed in H & E staining (Fig. 8C). This indicates the possibility of even tissue regeneration using the CNP GelMA. The ALP assay indicating higher ALP density for CNP GelMA hydrogel also suggests better bone regeneration potential (Fig. 8D). In the gene expression study, membrane bound glycoprotein ALP was also found to be higher in the CNP GelMA hydrogel-treated samples (Fig. 8E). Expression of mechano-sensing regulated marker (FAK) is also higher than CNP + GelMA hydrogel and similar to pure GelMA hydrogel. Type 1 collagen is significantly higher with all CNP-added hydrogel samples than pure GelMA hydrogel as observed from Col 1 upregulation. However, osteocalcin (OCN) gene expression was higher in pure GelMA hydrogel treated cells, though CNP GelMA hydrogel treated cells have better OCN expression than CNP + GelMA hydrogel treated cells.

## Discussion

The present research demonstrates the size and shape of the synthesized calcium phosphate nanoparticles can be modulated using the different concentrations of the gelatin polymer during its synthesis process. Initially Ca^++^ ions primarily interact with anionic hydroxyl, carboxyl and ester groups in the gelatin chemical structure and are expected to be distributed uniformly throughout its long polymeric chains (distribution). When PO_4_^3−^ is introduced into the system, insoluble calcium phosphates start forming (nucleation) and then grow. The polymeric chain entanglement density and physical crosslinking density of gelatin hinder the growth process of the particle leading to stabilizing the product into nanoparticles. If the amount of polymer chain is very high in the reaction media, the nanoparticles cannot grow freely to its regular crystal shape by the effect of higher entanglement density and crosslink density [32]. In the present study, we have observed similar interruption of the growth process of CNP with the increase in gelatin concentration in the reaction media. This not only influences the size of the synthesized nanoparticles, but also changes its shape significantly and gradually from spindle to spherical (Fig. 2A–D). Though the amount and size of nanoparticles in CNP (50%) GelMA is less than those of CNP GelMA, all are spherical in shape. This indicates that the amount of gelatin in the reaction media plays the major role in regulating the shape of the synthesized CNP. The concentrations of nanoparticle-forming agents are not important for shape modulation probably due to saturation of Ca^++^ binding sites in this study, but it gives the amount of synthesized nanoparticles and determines their sizes in the matrix. The overall reaction for size and shape modulation is suggested in the scheme provided as Fig. 2J. The extended conformation of gelatin polymer is expected from its dilute solution while the polymer chains would have entangled each other in higher concentrated solutions. The site for nucleation and growth to spindle shape is possible when the extended gelatin polymer chains are participating in the reaction medium. The growth process would be severely affected once the gelatin concentration is increased. The coiled or entangled morphology of polymer chains hinders the spindle-like growth during the nanoparticle formation and gradual change from spindle to spherical morphology is observed with increase in gelatin concentration in the reaction media as shown in Fig. 2K. Nanoparticle-forming reactants concentrations are important to determine the size of the nanoparticles, but the shape and aspect ratio are controlled by the polymeric chains of stabilizing gelatin matrix (provided sufficient time is given to complete the nucleation and growth process of the nanoparticles) (Fig. 2K).

The use of nanoparticles significantly reduces the free amine groups after methacrylation probably due to the interaction of CNP with amino group of gelatin through its PO_4_^3−^ group. The synthesis process adopted in this work suggested preoccupied carboxyl and hydroxyl groups with Ca^++^ ions. This may result in conformational changes in gelatin to make higher availability of free amine groups on the surface of the synthesized CNPs-gelatin complex as shown in Fig. 2J. The masking of other groups and availability of amine groups on surface of CNP GelMA due to this conformational change resulted in more methacrylation efficiency and subsequently faster photo-crosslinking than the CNP + GelMA. The methacrylation of gelatin creates new intense peaks as shown in the Fig. 3B [33].

Faster photo crosslinking for CNP GelMA can be attributed to better methacrylation of CNP GelMA (less availability of free amine groups) as evident from TNBS result (Fig. 3A). CNP + GelMA shows change in viscosity till 330 s. For the CNP GelMA, gelatin structure-based distribution of CNP coated with GelMA results its free amines to be exposed to outside homogeneously and have higher possibility to form methacrylation due to higher surface to volume ratio. CNP + GelMA may have localized aggregation and random distribution of CNPs in GelMA matrix. Further, the separately mixed nanoparticles may hinder the UV light penetration in the gel, resulting in more time requirement for crosslinking. The faster crosslinking process will help to crosslink the printed layers rapidly and will be controllable during 3D printing/bioprinting.

The cyclic compressive and print related mechanical properties of CNP GelMA outperform pure GelMA or CNP + GelMA. The control of hardness, adhesiveness, cohesiveness, and chewiness properties is necessary in the hydrogels for improved printability and mechanical properties in 3D bioprinting applications. The *in-situ* nanoparticle formation may induce more space in the gel structure leading to slightly higher swelling compared to separately mixed one. The self-standing height crosses 2 cm in the case of CNP GelMA which is significantly better height than the 3D bioprinted samples with various multi-component advanced gels reported by other researchers [34–42]. It can be concluded from the Figs. 4 and 5 results that the CNP GelMA can be successfully used for 3D printing/bioprinting with better structural stability. In this synthesis process, methacrylation is carried out subsequently in CNP-dispersed gelatin which makes the dispersion significantly better than the separately mixed CNP nanoparticles with GelMA polymer. After rigorous mixing using vortex shaking and ultrasonic treatment, the nanoparticle distribution is not uniform in the case of cross-linked CNP + GelMA hydrogel. This inhomogeneity can lead to uncontrolled tissue formation, which is a critical issue in its tissue engineering application for potential mechanical and biological properties in regenerated tissues.

The MTT assay (Supplementary Fig. 2) revealed biocompatible nature of all cross-linked hydrogel samples including the synthesized nanoparticle (CNP). Initially the samples with CNP showed less growth, however, within day 3, all samples showed more than 70% cell viability which is considered biocompatible [43]. However, separate mixing with nanoparticles leads to non-uniformity in bioactive nanoparticles distribution in the hydrogel matrix. This resulted in large variation in cell viability when it is mixed with GelMA solution separately with subsequent photo-crosslinking (Supplementary Fig. 2). Live and dead staining results confirmed that the bioactive ceramic nanoparticle helps to enhance the growth and proliferation of osteoblast cells. Homogenous distribution of nanoparticles and cell proliferations in our developed CNP GelMA adds additional advantage along with its superior mechanical and printing properties. The cell culture and gene expression studies using AdMSC cells are encouraging considering the even cell distribution and osteogenic differentiation possibilities using CNP GelMA. It is noteworthy that the AdMSC cells proliferate faster in CNP GelMA hydrogels and confluent in day 8 for 100 mm cell culture plate with 5000 cells seeding. Supplementary Fig. 3 shows the phase contrast images of the samples at the end of day 8. This indicates that the cell friendly atmosphere created by CNP GelMA hydrogel is significantly better than the cross-linked GelMA or a mixture of CNP and GelMA (CNP + GelMA) hydrogels.

## Conclusion

Amorphous calcium phosphates nanoparticles (CNP) have been synthesized through gelatin chemical structure-based calcium location and then phosphate addition-based nucleation and growth of CNP in the gelatin matrix as stabilizing agent through one pot processing. After the synthesis of nanoparticles, methacrylation of gelatin was carried out to synthesize nanoparticle reinforced GelMA (CNP GelMA). The nanoparticles’ size distributions and shape can be controlled depending upon the gelatin concentrations. Gelatin is used for nucleation and stabilization of nanoparticles; hence potential adverse effect of another stabilizing agent is minimized. Thus produced CNP GelMA shows significantly better mechanical and biological properties as well as printability than the separately mixed nanoparticles with GelMA upon photo-crosslinking. The photo-crosslinking becomes faster due to better methacrylation which is extremely important for continuous 3D bioprinting or printing of the tissue engineering construct. Almost three times increase in cyclic compressive properties (than pure GelMA hydrogel) and self-standing 3D printing height of more than 2 cm (160 printed layers) were achieved using crosslinked CNP GelMA hydrogel. MTT assay and live/dead staining using mouse osteoblast cells prove the biocompatible nature of the product upon cell encapsulation. The micro/nano-particle formation and morphologies in gelatin polymer prior to methacrylation are dependent upon gelatin concentrations. Easy and effective for gelatin’s chemical structure-based in situ nucleation and homogeneous distribution of micro/nanoparticles inside the gel matrix led to even in vitro tissue regeneration within short period. This CNP GelMA can be used as a better alternative to pure GelMA to overcome the structural stability issues during 3D printing or bioprinting of photo-crosslinked GelMA hydrogel and nanoparticle-incorporated prefabricated GelMA, not to mention of side effects of photo-crosslinkers such as longer time UV exposure and higher amount of crosslinkers.

## Supplementary Information

**Additional file 1: Figure 1.** (a) viscosity at shear rate 1 s^-1^ for different gels with different UV exposure time; Viscosity changes with shear rate for different UV exposure time of different gels, (b) GelMA, (c) CNP + GelMA, (d) CNP (50%) GelMA, and (e) CNP GelMA. **Figure 2.** MTT assay with MC3T3 cells for the hydrogels and CNP samples, where control is only medium. **Figure 3.** Phase contrast images on Day 8 of AdMSC cells cultured on Hydrogel samples for RT PCR study; (a) Control, (b) GelMA, (c) CNP + GelMA, (d) CNP GelMA. Table 1. The primer sequences used for qRT-PCR

**Additional file 2. **

## Data Availability

All data and materials are available upon request.

## Abbreviations

GelMA: Gelatin-methacryloyl
CNP: Calcium phosphate nanoparticle
DI: Deionized
SAED: Selected area electron diffraction
EthD-1: Ethidium homodimer-1
TNBS: 2,4,6-Trinitrobenzenesulfonic acid
EDS: Energy dispersive x ray
TGA: Thermogravimetric analysis
TEM: Transmission electron microscope
NMR: Nuclear magnetic resonance
ALP: Alkaline phosphatase
OCN: Osteocalcin
ATR-FTIR: Attenuated total reflectance Fourier-transform infrared spectroscopy
qRT-PCR: Quantitative reverse transcription-polymerase chain reaction
RNA: Ribose nucleic acid
DNA: Deoxyribose nucleic acid
FAK: Focal adhesion kinase
AdMSC: Adipose tissue derived mesenchymal stem cell
MTT: 3-(4,5-Dimethylthiazol-2-yl)-2,5-diphenyltetrazolium
PBS: Phosphate buffer saline
α-MEM: Minimum Essential Medium α
SEM: Scanning electron microscope
3D: Three dimensions
MC3T3: Osteoblast precursor cell line
