# Supplementary material for: Modulation of bioactive calcium phosphate micro/nanoparticle size and shape during in situ synthesis of photo-crosslinkable gelatin methacryloyl based nanocomposite hydrogels for 3D bioprinting and tissue engineering
Source: Biomater Res. 2022 Oct 8;26:54. doi: 10.1186/s40824-022-00301-6 (PMC9548207; doi:10.1186/s40824-022-00301-6)
Supplement: Supplementary file 1 — Additional file 1: Figure 1. (a) viscosity at shear rate 1 s-1 for different gels with different UV exposure time; Viscosity changes with shear rate for different UV exposure time of different gels, (b) GelMA, (c) CNP + GelMA, (d) CNP (50%) GelMA, and (e) CNP GelMA. Figure 2. MTT assay with MC3T3 cells for the hydrogels and CNP samples, where control is only medium. Figure 3. Phase contrast images on Day 8 of AdMSC cells cultured on Hydrogel samples for RT PCR study; (a) Control, (b) GelMA, (c) CNP + GelMA, (d) CNP GelMA. Table 1. The primer sequences used for qRT-PCR [file 40824_2022_301_MOESM1_ESM.docx]

**Modulation of bioactive calcium phosphate micro/nanoparticle size and shape during *in situ* synthesis of photo-crosslinkable gelatin methacryloyl based nanocomposite hydrogels for 3D bioprinting and tissue engineering**

Amitava Bhattacharyya ^1, 2, 3^, Gopinathan Janarthanan^1, 2^, Taeyang Kim^1^, Shiva Taheri^2^, Jisun Shin^1^, Jiyuan Kim^1^, Hyun Cheol Bae^4^, Hyuk-Soo Han^4^, Insup Noh ^1, 2*^

^1^ Department of Chemical and Biomolecular Engineering, Seoul National University of Science and Technology, Seoul 01811, Republic of Korea

^2^ Convergence Institute of Biomedical Engineering and Biomaterials, Seoul National University of Science and Technology, Seoul 01811, Republic of Korea

^3^ Functional, Innovative and Smart Textiles, PSG Institute of Advanced Studies, Coimbatore 641004, India

^4^ Department of Orthopedic Surgery, Seoul National University College of Medicine, Seoul

03080 Republic of Korea

***Correspondence: *insup@seoultech.ac.kr*

Submitted to *Biomaterials Research*

(August 12, 2022)


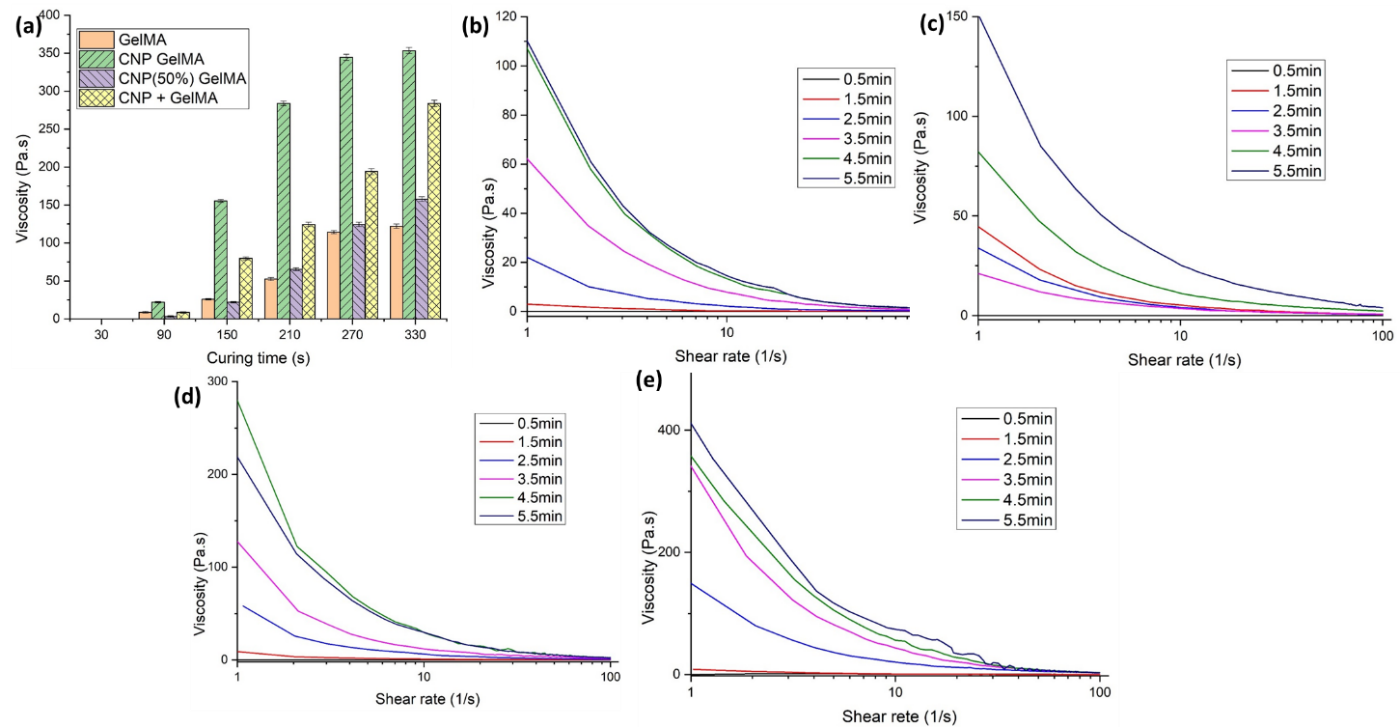


Figure 1: (a) viscosity at shear rate 1 s^-1^ for different gels with different UV exposure time; Viscosity changes with shear rate for different UV exposure time of different gels, (b) GelMA, (c) CNP + GelMA, (d) CNP (50%) GelMA, and (e) CNP GelMA


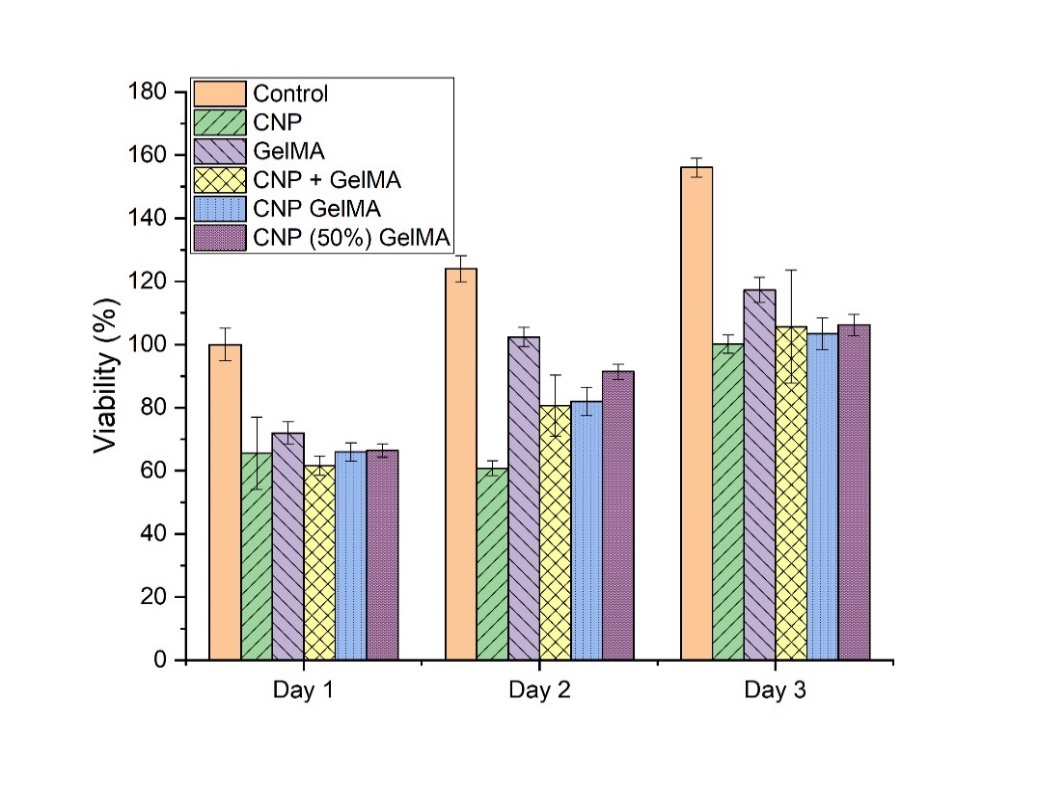


Figure 2: MTT assay with MC3T3 cells for the hydrogels and CNP samples, where control is only medium.


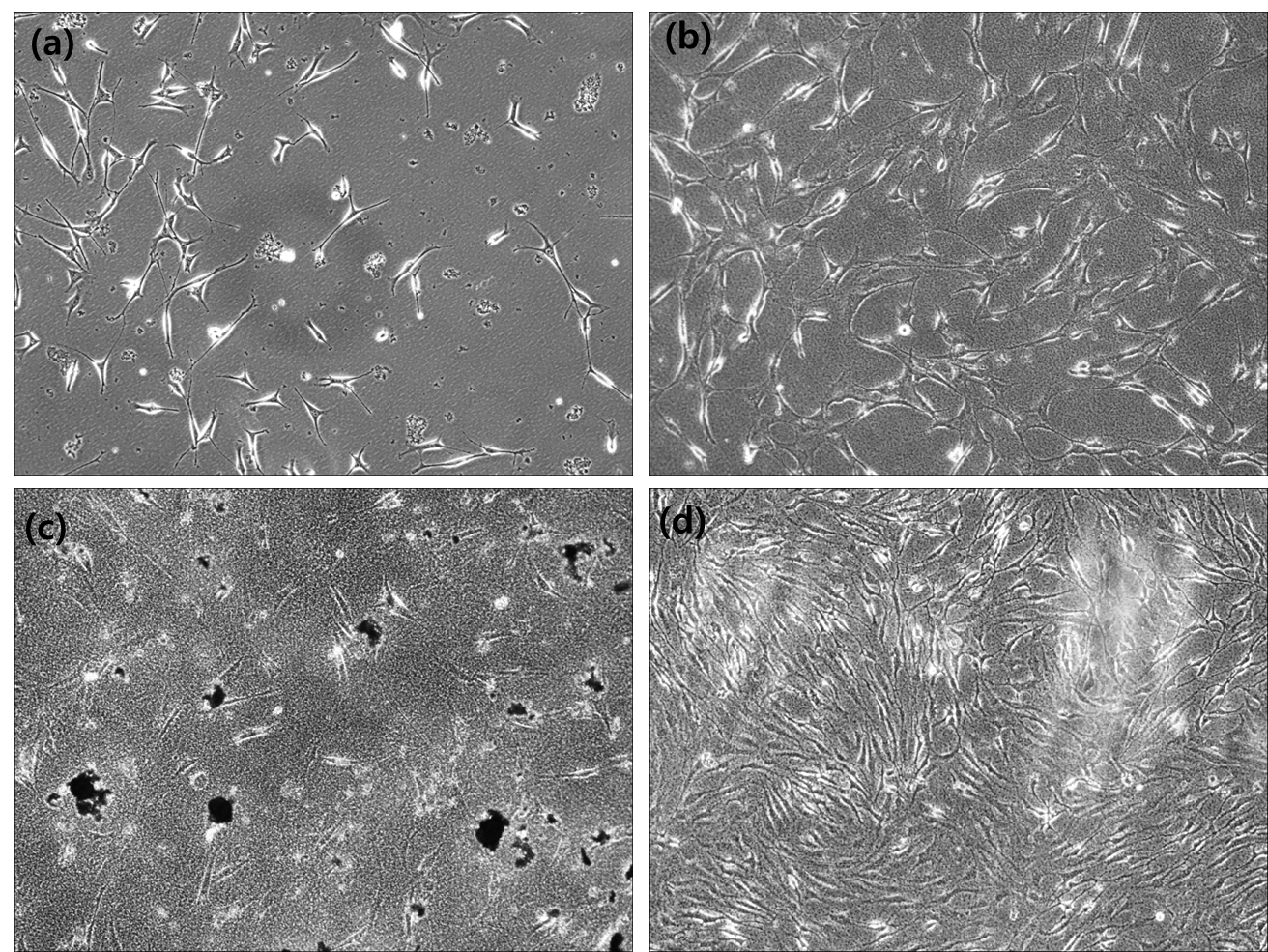


Figure 3: Phase contrast images on Day 8 of AdMSC cells cultured on Hydrogel samples for RT PCR study; (a) Control, (b) GelMA, (c) CNP + GelMA, (d) CNP GelMA.

**Table 1**. The primer sequences used for qRT-PCR

| Genes | Sequences |
| --- | --- |
| Col-1 | F: 5'-GACTTCTCCCAAGCGGGAAC-3' |
|  | R:5'-AGAAATTGAGCCCCAGGTTGA-3' |
| OCN | F: 5'-GAGGAAGTGGGCAGGAGAATG -3' |
|  | R: 5'-GTAGTAGAAAGGGGACAGGAC -3' |
| ALP | F: 5'-TGGACAAGTTCCCCTTCGTC-3' |
|  | R: 5'-AAAAGCCTCCAGAAGTGCCG -3' |
| FAK | F: 5'-ATGGCCTGCTATGGATTCTGC-3' |
|  | R:5'-TTGCGTCTTACTTCCACTCCT-3' |
| GAPDH | F: 5'-ATCCCATCACCATCTTCCAG-3' |
|  | R: 5'-CCATCACGCCACAGTTTCC-3' |
